# ZIF-8@Rhodamine B as a Self-Reporting Material for Pollutant Extraction Applications

**DOI:** 10.3390/nano13050842

**Published:** 2023-02-24

**Authors:** Edward W. P. Moore, Fernando Maya

**Affiliations:** Australian Centre for Research on Separation Science, School of Natural Sciences (Chemistry), University of Tasmania, Private Bag 75, Hobart, TSA 7001, Australia

**Keywords:** zeolitic imidazolate frameworks, rhodamine B, pollutant extraction, endocrine disrupting phenols

## Abstract

Herein, we have evaluated the potential of dye-encapsulation as a simple mechanism to self-report the stability of MOFs for pollutant extraction applications. This enabled the visual detection of material stability issues during the selected applications. As proof-of-concept, the zeolitic imidazolate framework (ZIF-8) material was prepared in aqueous medium and at room temperature in the presence of the dye rhodamine B. The total amount of loaded rhodamine B was determined using UV-vis spectrophotometry. The prepared dye-encapsulated ZIF-8 showed a comparable extraction performance with bare ZIF-8 for the removal of hydrophobic endocrine-disrupting phenols, such as 4-tert-octylphenol and 4-nonylphenol, and improved the extraction performance of more hydrophilic endocrine disruptors, such as bisphenol A and 4-tert-butylphenol.

## 1. Introduction

Metal–organic frameworks (MOFs) are porous crystalline materials synthesized from inorganic and organic units by strong bonds [1,2]. Due to the variety of available metals and organic building blocks (usually di- or polytopic organic carboxylates), thousands of different MOFs have been reported [2]. Applications of MOFs [3] and MOF composites [4] span different fields, such as catalysis [5], drug delivery [6], or energy storage and conversion [7]. Furthermore, there is a growing interest in the application of MOFs for environmental applications [8,9,10], such as the extraction [11] or catalytic degradation [12,13] of water pollutants. A well-known type of water-stable MOFs are zeolitic imidazolate frameworks (ZIFs) [14,15]. ZIFs are crystalline materials based on tetrahedrally coordinated metal ions connected by imidazole linkers with their resulting structures akin to zeolites. ZIFs are the subject of extensive research due to their highly microporous nature, facile formation from commercial precursors, and high thermal and chemical stability [16,17]. The structure and activity of the imidazole linker alongside the nodal metal ion combination determines the activity, morphology, surface area, and porosity of the ZIF crystal [17]. Therefore, the crystals formed can be tailored and tuned to specific applications via changing the imidazole linker present. Furthermore, ZIFs can also be used as precursors to obtain highly porous carbons for a range of applications [18,19].

Recent progress in the synthesis and application of ZIFs has been the subject of research to provide cost- and time-effective modes for the synthesis of such materials. The synthesis of ZIFs was first reported by Park et al. in 2006 [14] via solvothermal conditions in dimethylformamide with a ramping temperature gradient to 140 °C. Since then, the synthesis and progress of ZIFs has continued to expand to the room temperature aqueous synthesis [20] and even the solvent-free synthesis of ZIFs [21]. ZIF crystal growth in water at room temperature also enabled the encapsulation of guest molecules in the crystal structure, as reported for the biomimetic mineralization of biomacromolecules [22]. Aside from the direct encapsulation of proteins and enzymes in ZIFs [22], the encapsulation of other guest molecules, such as carbohydrates [23] or plasmids [24], has been also achieved. ZIFs also enabled the incorporation of small and polar organic molecules during crystal growth, as reported by the incorporation of dyes, such as rhodamine B [25], or the simultaneous encapsulation of multiple dyes [26].

Among the different potential applications for ZIFs, their application in the efficient removal of pollutants from water is promising [27], showing the potential of ZIFs in analytical applications [28] or environmental remediation [29]. Due to their excellent pollutant removal efficiency, MOFs could compete with other high-performing materials for the removal of pollutants from water [30,31]. However, a limitation for further development of this type of applications is the stability of ZIFs in certain mediums [32,33]. While ZIF-8 might be stable in pure water, stability of this material might be limited in other, more complex sample matrices, such as wastewater or seawater. An interesting example for monitoring of ZIF-8 stability in different mediums was developed by Kim et al. [34]. A three-dimensionally ordered macroporous mixed metal oxide ZnO-CeO_2_-Al_2_O_3_@ZIF-8 was used as a ZIF-8 precursor. ZnO provided the required Zn^2+^ ions for ZIF-8 growth on the support, and while the function of Al_2_O_3_ was structural, the presence of CeO_2_ enabled the monitoring of the stability of the ZIF-8 crystal structure. ZIF-8 stability monitoring was carried out by comparing changes in the relative X-ray powder diffraction (XRD) intensities between the ZIF-8 peak and the fully stable CeO_2_ peak. Incorporating stable metal oxide phases as internal standards allowed the precise evaluation of ZIF-8 stability but required the recovery of the crystals/support from the aqueous medium and the availability of an XRD instrument. Therefore, ZIF materials capable of self-reporting stability issues that are easily detected and do not require any instruments would be of interest.

**Figure 1:**
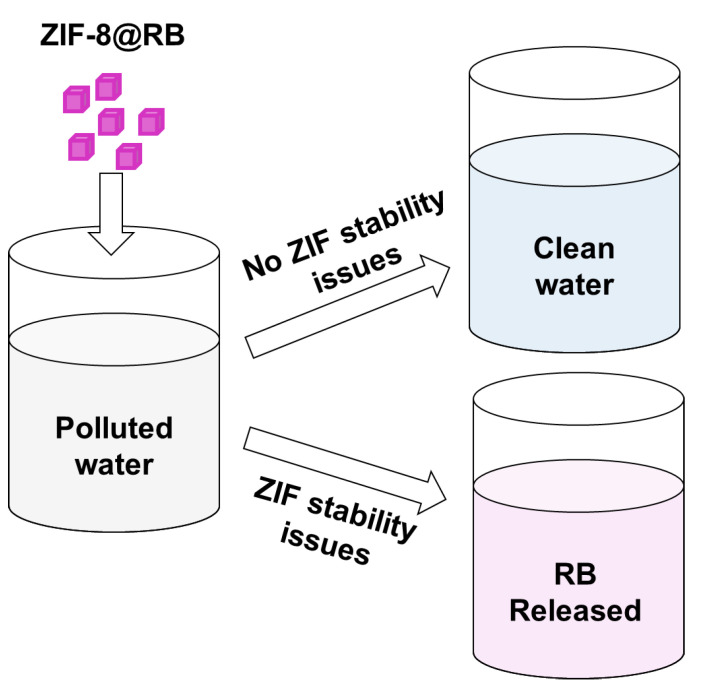
Scheme illustrating the self-reporting mechanism of ZIF-8@RB when stability issues are experienced during a pollutant removal application. RB, rhodamine B.

The aim of this work is the preparation of ZIF-8 with encapsulated rhodamine B (ZIF-8@RB) as a self-reporting material for the in situ visual detection of stability issues when the material is applied for pollutant extraction applications. Rhodamine B is released from ZIF-8 if the aqueous medium contains any substances that compromise the stability of the crystals (Figure 1). The effect of rhodamine B in ZIF-8 was evaluated for the extraction of different phenols (bisphenol A, 4-tert-butylphenol, 4-tert-octylphenol and 4-nonylphenol) used in plastic manufacturing, which are well known endocrine-disrupting compounds [35]. These pollutants can mimic the action of endogenous steroid hormones in living organisms, potentially influencing the development of wildlife and humans. This is the first example of an extraction application using MOFs, enabling visual stability monitoring of the extraction material.

## 2. Materials and Methods

### 2.1. Reagents 

Zinc nitrate hexahydrate (98%), 2-methylimidazole (99%), sodium hydroxide (98%), methanol (HPLC grade, 99.9%), ethylenediaminetetraacetic acid (99%), sodium chloride (99%), potassium dihydrogen phosphate (99%), bisphenol-A (97%), 4-tert-butylphenol (99%), 4-tert-octylphenol (97%), and 4-nonylphenol (99%), were purchased from Sigma-Aldrich. Rhodamine B (≥95%) was purchased from VWR. Potassium chloride (99.8%) was purchased from AJAX Chemicals. Disodium hydrogen phosphate (99%) was purchased from Merck, Rahway, NJ, USA. 

### 2.2. Instrumentation

The morphology of the prepared materials was studied using a Hitachi SU-70 field emission scanning electron microscope (FESEM) at an accelerating voltage of 1.5 KV. Samples were prepared via dispersing the ZIF-8 crystals in methanol. A droplet of the resulting dispersion was added onto a glass slide atop titanium pegs and methanol was evaporated. XRD measurements were carried out using a benchtop Bruker D2 phaser diffractometer with a Co X-ray anode and adjusted to correct for difference between Cu and Co anodes. A Micromeritics Tristar II surface area and porosity analyzer was used to measure the nitrogen adsorption–desorption isotherms of the prepared materials. The Brunauer–Emmett–Teller (BET) method was used to calculate the surface area using experimental points at relative pressures between P/P_0_ = 0.05–0.3. Samples were pretreated by heating under vacuum at 90 °C for 1 h, then at 140 °C for 16 h. Ultraviolet-visible spectrophotometry (UV-vis) was used to quantify the release of rhodamine B from the ZIF-8 crystals.

An HPLC (Agilent 1290 Infinity) equipped with an Agilent 1290 binary pump, an automatic Agilent 1290 sampler, and an Agilent 1290 DAD detector was used for pollutant quantification after extraction with the prepared materials. A Supelco Nucleosil 100-5 C18 column (150 × 4.6 mm I.D., 5 μm packing particle size, Merck KGaA, Darmstadt, Germany) equipped with a guard column (10 × 4.6 mm I.D., 5 μm packing particle size, Merck) of the same material was used to separate the analytes. The mixture of the selected pollutants in aqueous medium was separated using gradient elution with a mobile phase consisting of 65% methanol/35% water for 1 min, and increasing to 75% methanol at 5 min, 85% methanol at 10 min, 95% methanol at 20 min, and decreasing to 65% methanol at 22 min. Separations were carried out at room temperature using a flow rate of 1 mL min^−1^. Analyte detection was performed at 229 nm, and the injected sample volume was 10 µL. All ZIF stability and pollutant extraction experiments were carried out in triplicate.

### 2.3. Synthesis of ZIF-8

The procedure for the aqueous synthesis of ZIF-8 was based on a procedure reported in the literature [26] with modifications. Zinc nitrate hexahydrate (0.4615 g) was dissolved in 25 mL of water. A solution of 2-methylimidazole (11.36 g) was dissolved in 175 mL of water. The 2 aqueous solutions were combined and stirred for 10 s and left to crystallize for 24 h at room temperature. The resulting white crystals were centrifuged down at 4500 rpm for 5 min and washed with water (4 × 30 mL) and methanol (4 × 30 mL) to remove the excess unreacted precursors. The crystals were then transferred to an oven at 60 °C and left to dry overnight. For the synthesis of ZIF-8@RB, the same procedure was followed, but by dissolving rhodamine B into the 2-methylimidazole solution to a resulting concentration of 0.05, 0.1, 0.25, 0.5, 0.75, 1.0, or 2.5 g L^−1^ rhodamine B.

### 2.4. Determination of Rhodamine B-Loading in ZIF-8 via UV-Vis Spectrophotometry

An aqueous stock solution of rhodamine B (0.1 g L^−1^) was prepared by dissolving rhodamine B (0.01 g) on 100 mL Milli-Q water. A calibration curve was carried out by stepwise dilution of the stock solution, covering the range between 1 mg L^−1^–5 mg L^−1^ rhodamine B. The ZIF-8@RB crystals (5 mg) prepared in the presence of different amounts of rhodamine B were dissolved in a solution of ethylenediaminetetraacetic acid (EDTA) (0.1 mol L^−1^, 5 mL). These solutions were placed in an orbital shaker oscillated until full dissolution of the crystals had occurred. The resulting solutions were then analyzed using UV-Vis spectrophotometry to determine the concentration of rhodamine B released from the dissolved crystals. For the case of ZIF-8 prepared using a 2-methylimidazole solution containing 2.5 g L^−1^ rhodamine B, the released rhodamine B was too concentrated and thus a 10-fold dilution took place so that the values presented were within the measurable range of the UV-Vis and calibration curve. 

### 2.5. Buffers and Salts

Four different buffer and salt solutions were prepared to compare the stability of ZIF-8@RB in different mediums:

(1) Phosphate buffer solution (PBS) 1.0 M. Sodium chloride (8 g, 0.137 mol L^−1^), potassium chloride (0.2 g, 2.7 mmol L^−1^), sodium hydrogen phosphate (1.77 g, 10 mmol L^−1^), and potassium dihydrogen phosphate (0.24 g, 1.8 mmol L^−1^), were dissolved in 800 mL of Milli-Q water. The solution was adjusted to pH 7.4 using NaOH (1 mol L^−1^) and diluted to a final volume of 1 L with Milli-Q water.
(2) Phosphate buffer solution 0.1× (PBS 0.1×). 10 mL of PBS was diluted using Milli-Q water to a volume of 100 mL to obtain a 0.1 M solution of PBS.
(3) Ethylenediaminetetraacetic acid (EDTA). EDTA (2.92 g, 0.1 M) was dissolved in 100 mL of Milli-Q water.
(4) Sodium chloride (NaCl). A 1.0 M solution of sodium chloride was prepared by dissolving 5.844 g NaCl into 100 mL of distilled water.

ZIF-8@RB (10 mg) was added to the above solutions (10 mL) and the absorbance of the solution was measured at 554 nm after 24 h.

### 2.6. Mixed Phenol Extraction 

Individual phenol stock solutions were prepared by the addition of bisphenol-A, 4-tert-butylphenol, 4-tert-octylphenol, and 4-nonylphenol (10 mg) into separate containers followed by the addition of methanol (10 mL) obtaining 1000 mg L^−1^ solutions of each one of the phenols. From this solution, serial dilutions were undertaken wherein 1 mL of each solution was diluted up to 100 mL with water to form a 10 mg L^−1^ solution of each phenol. A volume of 10 mL of each one of these solutions was combined in a 100 mL volumetric flask, resulting in a 1 mg L^−1^ aqueous mixed phenol standard solution. Separate experiments were conducted by adding either ZIF-8 (10 mg) or ZIF-8@RB (10 mg) to the aqueous mixed phenol standard solution (1 mg L^−1^, 10 mL), and these were left in an orbital shaker for 24 h. The resulting mixtures were filtered through a syringe filter (0.45 µm pore size) and the concentrations of the different phenols remaining in the aqueous solution were measured using HPLC.

## 3. Results and Discussion

### 3.1. Screening of Rhodamine B Concentration in ZIF-8 Synthesis

**Figure 2:**
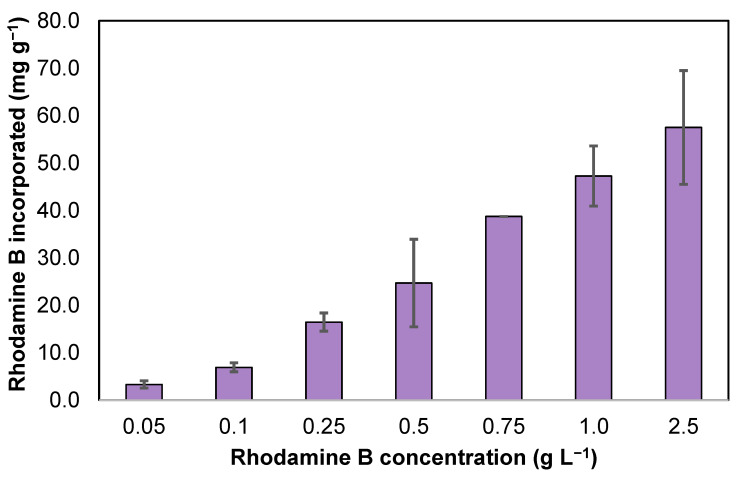
Effect of initial rhodamine B concentration on the amount of rhodamine B incorporated on the obtained solid products.

ZIF-8 was prepared in aqueous solutions containing different concentrations of rhodamine B, and a method to monitor the amount of rhodamine B encapsulated in the obtained crystals was developed. The aim of this preliminary screening is to obtain the ZIF-8 with the highest rhodamine B uptake. In order to be able to quantify the amount of rhodamine B in the ZIF-8 crystals, a calibration curve of rhodamine B in water was carried out using UV-vis spectrophotometry in the range of 1–5 mg L^−1^ of rhodamine B (y = 0.1848x − 0.0027; R^2^, 0.9996). Rhodamine B was added to the 2-methylimidazole aqueous solutions to a resulting concentration of 0.05, 0.1, 0.25, 0.5, 0.75, 1.0, 2.5 g L^−1^. To examine the amount of rhodamine B encapsulated within each of the synthesized solids, an experiment was undertaken wherein 5 mg of each solid sample was placed in a solution of EDTA (0.1 mol L^−1^, 5 mL). The resulting solutions were placed in an orbital shaker until the solids have completely dissolved. EDTA was employed for dissolution of the resulting Zn(II)/2-methylimidazole-based solids as the EDTA binds to the zinc as a chelating ligand and replaces the 2-methylimidazole, forming a water-soluble Zn-EDTA complex. The absorbance of the released rhodamine B was then measured using UV-vis spectrophotometry. Figure 2 shows a clear trend regarding an increasing amount of rhodamine B incorporated into the obtained precipitates based on Zn(II) and 2-methylimidazole, while increasing the concentration of rhodamine B in the synthesis step up to a concentration of 2.5 g L^−1^.

The yields for the prepared materials were compared with the yield obtained by using Zn(II) and 2-methylimidazole in the absence of rhodamine B, which produced ZIF-8 microcrystals with a rhombic dodecahedron morphology, as characterized in the next section. The yield of the obtained products in the presence of rhodamine B was comparable to pure ZIF-8 for concentrations of up to 0.25 g L^−1^, obtaining yields >75%. Using higher concentrations of rhodamine B led to a drastic decrease in the yield of the obtained products (Yield, 5–25%). In a compromise between the yield obtained and the amount of rhodamine B incorporated in the material, the product obtained using a concentration of 0.25 g L^−1^ of rhodamine B was further characterized.

### 3.2. Characterization of ZIF-8@RB

**Figure 3:**
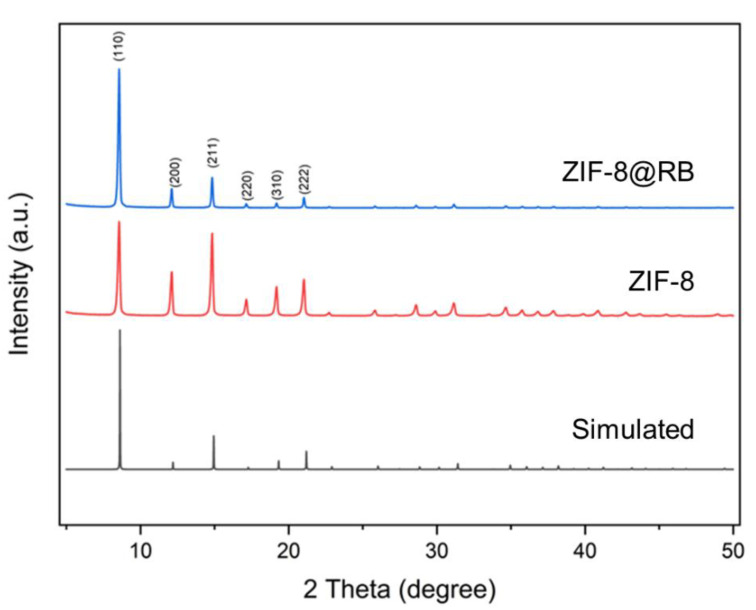
XRD patterns of ZIF-8, ZIF-8@RB and simulated ZIF-8.

Herein, XRD was carried out to determine if the ZIF-8 synthesized in the presence of rhodamine B had maintained the original ZIF-8 crystalline structure. The XRD patterns of ZIF-8 and ZIF-8@RB are shown in Figure 3. The ZIF-8@RB in this study was prepared by adding rhodamine B to the 2-methylimidazole aqueous solutions, resulting in a concentration of rhodamine B of 0.25 g L^−1^. For both materials the main six facet planes matched at 2 Theta = 7.55, 12.11, 14.83, 17.13, 19.18, 21.03 degrees which correspond to the facet planes of (110), (200), (211), (220), (310), and (222), respectively. These facets are characteristic of ZIF-8 as is confirmed by the simulated XRD pattern of ZIF-8 (Figure 3). 

**Figure 4:**
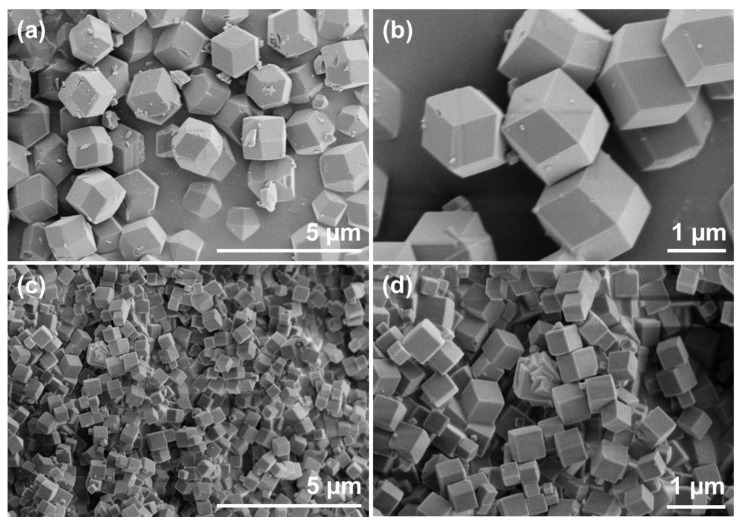
SEM images of ZIF-8 (**a**,**b**) and ZIF-8@RB (**c**,**d**).

To investigate the morphology of the ZIF-8 and ZIF-8@RB and the effect of the addition of rhodamine B on the crystal structure of ZIF-8@RB, SEM was employed. Figure 4a,b, show the morphology of the ZIF-8 crystals at different magnifications. ZIF-8 crystals display a rhombic dodecahedron morphology with crystal sizes ranging from 1–2 µm. The morphology of the synthesized ZIF-8 is confirmed to be rhombic dodecahedron and its sizing is consistent with methodologies which share the zinc nitrate hexahydrate starting reagent [26,36]. SEM images of ZIF-8@RB (prepared using a concentration of 0.25 g L^−1^ rhodamine B) at different magnifications are shown in Figure 4c,d. Compared to ZIF-8, ZIF-8@RB had smaller crystals with a submicrometric crystal size range. The size of most crystals was between 0.5 µm–0.7 µm and presented a cubic morphology but with truncated edges. This change in morphology might be attributed to the effect of rhodamine B slowing down the growth of the ZIF-8 crystals as the rhombic dodecahedron with truncated corners shape is one of the different morphologies the ZIF-8 crystal takes on before reaching its final rhombic dodecahedron shape.

**Figure 5:**
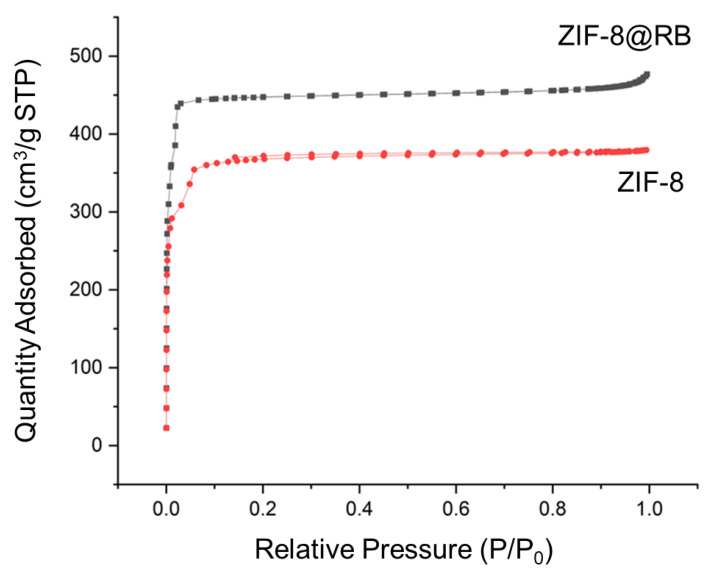
Nitrogen adsorption–desorption isotherms of ZIF-8 and ZIF-8@RB.

Nitrogen adsorption/desorption porosimetry was employed to characterise both ZIF-8 and ZIF-8 rhodamine B materials and to observe any porosity changes presented by the encapsulation of rhodamine B. The prepared ZIF-8 crystals had a surface area of 1392 m^2^ g^−1^ and a type I isotherm with a steep nitrogen uptake increase at P/P_0_ < 0.1, typical of microporous materials (Figure 5). The obtained surface area for ZIF-8 falls in the expected range normally reached when this material is prepared in aqueous medium and at room temperature. A significant increase in surface area was measured for the ZIF-8@RB with a surface area of 1790 m^2^ g^−1^ (Figure 5), in agreement with other preparations of ZIF-8@RB already reported in the literature [25]. ZIF-8@RB also presented a Type I isotherm but, in this case, a small increase in nitrogen uptake is also observed for P/P_0_ > 0.9, which is attributed to the potential formation of interparticle macropores due to the aggregation in the dry state of the smaller size of the ZIF-8@RB crystals. 

### 3.3. UV-Vis Spectrophotometry Monitoring of ZIF-8@RB Stability in Different Media

**Figure 6:**
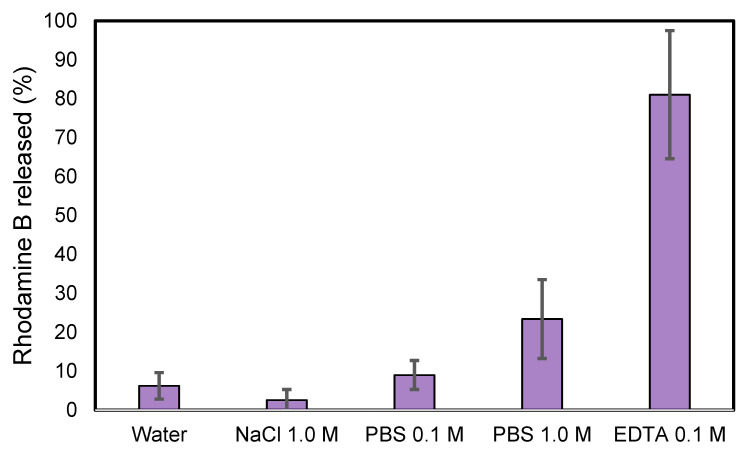
Stability of ZIF-8@RB in different mediums.

The stability of ZIF-8@RB in different media, such as salts and buffers, was evaluated. The methodology consisted of placing the ZIF-8@RB (10 mg) in 10 mL of each of the prepared media solutions (See Section 2.5) and leaving the solutions in an orbital shaker for 24 h. The percentage of rhodamine B released was measured after the 24 h and this experiment was repeated in triplicate. Figure 6 shows that ZIF-8@RB is unstable in 0.1 M EDTA due to the chelating effect of the EDTA binding the Zn(II) of ZIF-8. In all instances, a release of at least 65% of the rhodamine B amount present in ZIF-8 was observed, confirming the instability of ZIF-8 in this medium. Performing the same experiment in water, or in NaCl 1 mol L^−1^ solution, an average value <6% of the rhodamine B present in the ZIF-8 material was released after 24 h. This could be attributed to a residual release of rhodamine B because of the extended soaking times of the ZIF-8@RB crystals in these solutions, since no residual leaching was observed in the last washing steps after ZIF-8@RB preparation using water or methanol. While rhodamine B release was <10% when using PBS 0.1 M as the medium, a significant increase was observed using a more concentrated PBS buffer of 1.0 M, producing a 22% release of rhodamine B, confirming the limited stability of ZIF-8 depending on the concentration of PBS buffer used. These results confirmed the usability of rhodamine B release as a simple and fast approach to detect potential stability issues in the application of MOFs for environmental applications. However, to know exactly the details of the mechanism of this instability, full characterization of the crystalline material after extraction would be required.

### 3.4. Simultaneous Extraction of Phenols from Water

The performance of ZIF-8 and ZIF-8@RB for organic pollutant extraction was then compared. The organic pollutants selected for this study were phenols, specifically bisphenol-A, 4-tert-butylphenol, 4-tert-octylphenol, and 4-nonylphenol. These phenols were chosen due to their persistence in the environment in natural waterways and soil near waterways alongside their toxicity [37]. Thus, an experiment was undertaken wherein ZIF-8 (10 mg) or ZIF-8@RB (10 mg) were placed into 1 mg L^−1^ standard solution containing both bisphenol-A, 4-tert-butylphenol, 4-tert-octylphenol, and 4-nonylphenol. These solutions were placed in an orbital shaker for 24 h. The resulting mixtures were filtered by syringe filters (0.45 µm pore size) to filter the ZIF-8 and ZIF-8@RB crystals and the resulting filtrate was analyzed by HPLC-UV to quantify the remaining phenols in the solution and thus the amount extracted by the ZIFs. 

**Figure 7:**
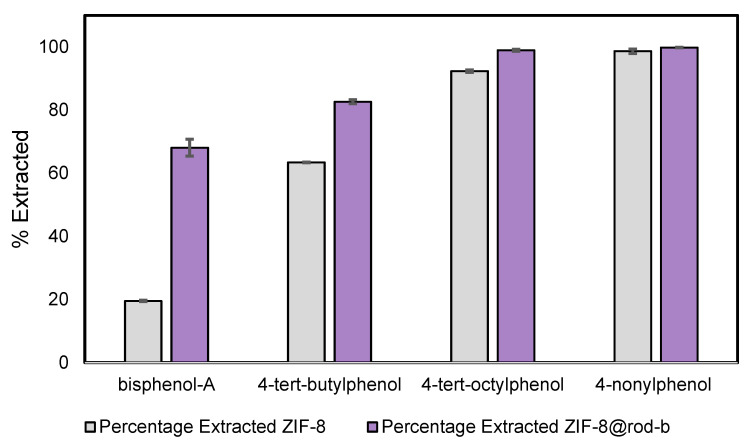
Performance of ZIF-8 and ZIF-8@RB for the extraction of phenols from water.

The percentage of phenols extracted by the ZIF materials is shown in Figure 7, which highlights both the similarities and the differences between ZIF-8 and ZIF-8@RB in the simultaneous extraction of these organic pollutants. Regarding the two most hydrophobic phenols, a high removal percentage for both ZIF-8 and ZIF-8@RB under the selected experimental conditions was observed. 4-nonylphenol removal was 98.9% with the ZIF-8@RB and 98.6% with ZIF-8. 4-tert-octylphenol removal was slightly higher with the ZIF-8@RB (99.8%) compared to ZIF-8 (92.3%). For the more polar pollutants, bisphenol-A and 4-tert-butylphenol, there were significant differences in percentage removal when comparing ZIF-8@RB with ZIF-8. The ZIF-8@RB showed a greater extraction performance for both these phenols at 68% and 82% removal for bisphenol A and 4-tert-butylphenol, whereas the ZIF-8 has a lower extraction for these phenols at 19 % and 63 %, respectively. This effect could be attributed to additional interactions established between the rhodamine B present in the ZIF-8 crystals and the selected pollutants. However, additional studies would be required to confirm the nature of these interactions. Another factor involved here is the effect of rhodamine B in the different size and shape of the ZIF-8@RB compared to bare ZIF-8, which could also influence pollutant mass transfer, and therefore, the percentage removal of these pollutants. ZIFs could be a potential alternative for combining with other efficient and cost-effective materials, such as layered double hydroxides (LDH) [38,39]. While LDHs have a high performance in the extraction of metal pollutants, ZIFs provide a high efficiency for the extraction of pollutants of an organic nature.

## 4. Conclusions

The encapsulation of rhodamine B within ZIF-8 under specific synthesis conditions in aqueous medium and at room temperature has been documented and studied in the formation of ZIF-8@RB. The incorporation of a dye in the ZIF-8 crystals for their subsequent use as sorbents for the removal of organic pollutants from water showed the following advantages: (1) dye release when ZIF crystals are unstable in the sample matrix enables the simple visual detection of stability issues during the extraction process; (2) the presence of rhodamine B in ZIF-8 showed improved removal of polar pollutants and simultaneously maintained the high performance in the removal of less-polar pollutants, as exemplified with a mixture of endocrine disrupting phenols; (3) ZIF-8@RB is synthesized in aqueous medium from available and relatively cheap precursors (zinc nitrate, 2-methylimidazole, and rhodamine B), which could facilitate potential commercial viability. 

Further research that would help to bolster the understanding of this crystalline ZIF-8@RB material would be a full characterization study of the role of rhodamine B in the material and its contribution to further increase the surface area of ZIF-8. It would be beneficial to create a comprehensive library of stability media to better understand the degradation conditions of the ZIF-8 and ZIF-8@RB, such as solutions of different pH, more concentrated or diluted salt solutions, or solutions of more complex matrices with known compositions such as simulated seawater, wastewater, or urine. The scope of application for the ZIF-8@RB material could be also expanded in the future into the preparation of membranes for online continuous flow extraction.

## Data Availability

Not applicable.
